# Validation of the fullPIERS model for predicting severe maternal outcomes in preeclampsia in five Brazilian centers

**DOI:** 10.1002/ijgo.70989

**Published:** 2026-04-02

**Authors:** Pedro do Valle Teichmann, José Paulo Siqueira Guida, Maria Laura Costa, Leila Katz, Melania Maria Amorim, Jussara Mayrink, Giorgio Tondello, Sergio Hofmeister de Almeida Martins Costa, José Geraldo Lopes Ramos

**Affiliations:** ^1^ Federal University of Rio Grande do Sul Porto Alegre Brazil; ^2^ University of Campinas Campinas Brazil; ^3^ Brazilian Network for Studies in Hypertension in Pregnancy Botucatu Brazil; ^4^ Instituto de Medicina Integral Prof. Fernando Figueira (IMIP) Recife Brazil; ^5^ Federal University of Minas Gerais Belo Horizonte Brazil; ^6^ Darcy Vargas Maternity Joinville Brazil

**Keywords:** decision support techniques, maternal mortality, preeclampsia, receiver operating characteristic curve, risk assessment

## Abstract

**Objective:**

The objective of this study is to validate the fullPIERS (Pre‐eclampsia Integrated Estimate of Risk) prognostic model in a large, multicenter cohort of Brazilian women with preeclampsia and to determine the optimal cutoff for clinical application in the Brazilian context.

**Methods:**

This was a secondary analysis of five prospective Brazilian studies evaluating the fullPIERS model, conducted in tertiary obstetric referral centers across three geographic regions (Porto Alegre, Campinas, Recife, Belo Horizonte, and Joinville). A total of 1581 pregnant women with confirmed preeclampsia were included. The primary outcome was the occurrence of one or more severe maternal complications within 48 h of assessment. FullPIERS risk estimates were calculated using routinely collected clinical and laboratory variables. Model performance was assessed by receiver operating characteristic (ROC) curve analysis.

**Results:**

Severe maternal complications occurred in 235 women (14.86%), with HELLP (hemolysis, elevated liver enzymes and low platelets) syndrome being the most frequent (9.5%). The fullPIERS model demonstrated moderate discriminative ability, with an area under the ROC curve of 0.753 (95% confidence interval: 0.714–0.791). The optimal cutoff value of 2.35% yielded a sensitivity of 58.3%, specificity of 84.6%, and a negative predictive value (NPV) of 94.8%.

**Conclusion:**

In this large, geographically diverse Brazilian cohort, the fullPIERS model showed substantial clinical utility for ruling out severe maternal complications in women with preeclampsia. Its high NPV supports its use in referral decision‐making and prioritization of resources. Given its reliance on routinely available clinical data, fullPIERS might serve as a valuable tool to optimize maternal care pathways, particularly in resource‐limited settings.

## 1 INTRODUCTION

Hypertensive disorders of pregnancy are a major contributor to maternal mortality, corresponding to 16% of all maternal deaths globally, and 22% in Latin America.^1^ In Brazil, preeclampsia (PE) affects 6.7% of pregnant women, and its complications are the leading cause of maternal death in the country.^2^ Beyond this, PE accounts for a significant proportion of preterm births.^3^ Timing of birth is key in the management of PE cases; however, the decision to prioritize maternal benefit through pregnancy termination versus the risks of prematurity is often challenging.

Low‐ and middle‐income countries like Brazil face daily constraints in terms of the number of beds available for maternal care and neonatal intensive care units (NICUs) and the need to transfer women to centers where specialized care is available. This all takes time, and the medical team must balance the risks of prematurity and maternal deterioration.^4^ Therefore, tools that support clinical teams in identifying cases that can be managed without immediate delivery are essential, thereby reducing preterm birth rates while preserving maternal health by identifying those at higher risk of clinical deterioration in a short timeframe.^5^, ^6^

In this context, strategies to estimate the risk of maternal complications have been proposed, including the use of algorithms or biomarkers. The fullPIERS (Pre‐eclampsia Integrated Estimate of Risk) is a validated prognostic model designed to predict severe maternal complications related to PE within 48 h. This model was proposed considering a range of adverse outcomes that include maternal death, HELLP syndrome (hemolysis, elevated liver enzymes and low platelets), eclampsia, placental abruption, renal failure, pulmonary edema, the need for intubation or blood transfusion, elevated liver enzymes, and neurological complications.^7^

The model uses clinical and laboratory variables routinely collected in clinical practice to estimate the risk of these severe maternal outcomes. These variables include gestational age, presence of dyspnea or chest pain, oxygen saturation, and creatinine, liver transaminases, and platelet count. Due to the complexity of the equation, free online calculators are available to estimate the probability of severe maternal outcomes.^7^ However, there are no established cutoff points to determine interventions, a fundamental aspect for clinical implementation, requiring the cutoff to be adapted according to local epidemiological characteristics and resource availability.

The objective of this study is to validate the use of the fullPIERS model using databases from specialized centers in different regions of Brazil, to estimate the optimal cutoff point with the best accuracy for clinical use in the Brazilian context.

## 2 METHODS

### 2.1 Study design

This was a secondary analysis of five Brazilian studies conducted in the cities of Porto Alegre, Campinas, Recife, Belo Horizonte, and Joinville. The first four original studies have already been published.^8^, ^9^, ^10^, ^11^ This study followed the Standards for Reporting of Diagnostic Accuracy Studies (STARD) guidelines for diagnostic accuracy research.^12^

### 2.2 Study settings

All included centers are public referral hospitals for the care of high‐risk pregnancies, with full obstetric and anesthesia teams and availability of intensive care beds for both pregnant women and newborns.

The study included two centers from the Southern region (Hospital de Clínicas de Porto Alegre – HCPA, in Porto Alegre, and Maternidade Darcy Vargas, in Joinville), one from the Northeastern region (Instituto de Medicina Integral Prof. Fernando Figueira – IMIP, in Recife), and two from the Southeastern region (University of Campinas Women's Hospital – UNICAMP, in Campinas, and the Hospital das Clínicas of the Federal University of Minas Gerais – HC‐UFMG, in Belo Horizonte).

### 2.3 Study period

Data collection periods varied across centers: at HCPA, data were collected from January 2017 to August 2024; at Maternidade Darcy Vargas, from January 2020 to December 2023; at IMIP, from January to December 2014; at UNICAMP, from January 2017 to February 2018; and at HC‐UFMG, from January 2014 to December 2023.

### 2.4 Study procedures

During the 2025 Brazilian Congress of Gynecology and Obstetrics, researchers who had conducted studies using the fullPIERS were invited to collaborate with a national database on fullPIERS if the following criteria were adopted during original data collection.

The centers must have included all pregnant women diagnosed with PE or superimposed PE during the respective study periods who had complete data available to allow retrospective calculation of the fullPIERS score at the time of hospital admission.

The inclusion criteria were the presence of PE or superimposed PE. PE was defined as new‐onset hypertension (systolic ≥140 mmHg and/or diastolic ≥90 mmHg) after 20 weeks of gestation, with or without proteinuria, associated with maternal organ dysfunction or signs of uteroplacental insufficiency. Superimposed PE was defined as chronic hypertension with new or worsening proteinuria, increased need for antihypertensive therapy, or signs of end‐organ dysfunction.^13^

Chronic hypertension was defined as the presence of hypertension prior to 20 weeks of pregnancy, with or without the need for antihypertensive medication. We also considered as chronic hypertension those women who were in use of antihypertensive medication prior to pregnancy.^13^

Organ dysfunction was diagnosed by clinical or laboratory criteria. Clinical criteria were considered when headache, abdominal pain, pulmonary oedema, or visual symptoms were present. The presence of one or more of the following laboratory findings defined organ dysfunction: creatinine >1 mg/dL, transaminases >70 U/L, and platelet count <150 000/mL. Uteroplacental insufficiency occurred when fetal growth restriction or abnormal umbilical artery Doppler were detected.^13^

Women with autoimmune diseases, or other chronic conditions such as diabetes, chronic kidney disease, or systemic lupus erythematosus, were also included if they met the abovementioned inclusion criteria.

Exclusion criteria were multiple gestations, known fetal malformations, confirmed congenital infections, or a diagnosis of PE made only in the postpartum period.

The fullPIERS score was calculated retrospectively using the original model variables: gestational age, presence of chest pain or dyspnea, peripheral oxygen saturation, platelet count, serum creatinine, and aspartate aminotransferase. Data were extracted from electronic medical records and entered into the online fullPIERS calculator (https://pre‐empt.obgyn.ubc.ca/home‐page/past‐projects/fullpiers/).

The primary outcome was the occurrence of maternal death, HELLP syndrome, eclampsia, placental abruption, renal failure, pulmonary edema, the need for intubation or blood transfusion, elevated liver enzymes, neurological complications, or any maternal complication during hospitalization.

Mode of delivery was not required for inclusion in the analysis, as it is not part of the fullPIERS model nor of the primary outcome definition. The primary outcome was based exclusively on maternal complications occurring during hospitalization.

### 2.5 Statistical analysis

The researchers who performed studies following the abovementioned criteria, and who agreed to share their database, sent the database for statistical analysis. The five databases were combined into one database, and internal consistency was performed to identify possible missing or inconsistent data. The local center researcher resolved those identified inconsistencies.

Descriptive statistics were presented as medians and interquartile ranges (IQR) for continuous variables and as absolute and relative frequencies for categorical variables. The discriminatory performance of the fullPIERS score was assessed through receiver operating characteristic (ROC) curves. The area under the curve (AUC) of the ROC curve, with 95% confidence intervals (CI), was calculated for combined adverse maternal outcomes. The Youden index was calculated to identify the cutoff point that provided the best balance between sensitivity and specificity.

### 2.6 Ethical evaluation

For participating in this study, all centers presented the local Institutional Review Board approval for the original research. Written consent was obtained from each participant before inclusion in the research.

## 3 RESULTS

A total of 1581 women were included in the study: 661 (41.8%) from Porto Alegre, 299 (18.9%) from Recife, 208 (13.1%) from Campinas, 196 (12.4%) from Belo Horizonte, and 217 (13.7%) from Joinville. Figure 1 shows the geographic distribution of the centers on the map of Brazil. There were 235 severe maternal outcomes recorded in the sample, resulting in a prevalence of 14.86%.

**FIGURE 1:**
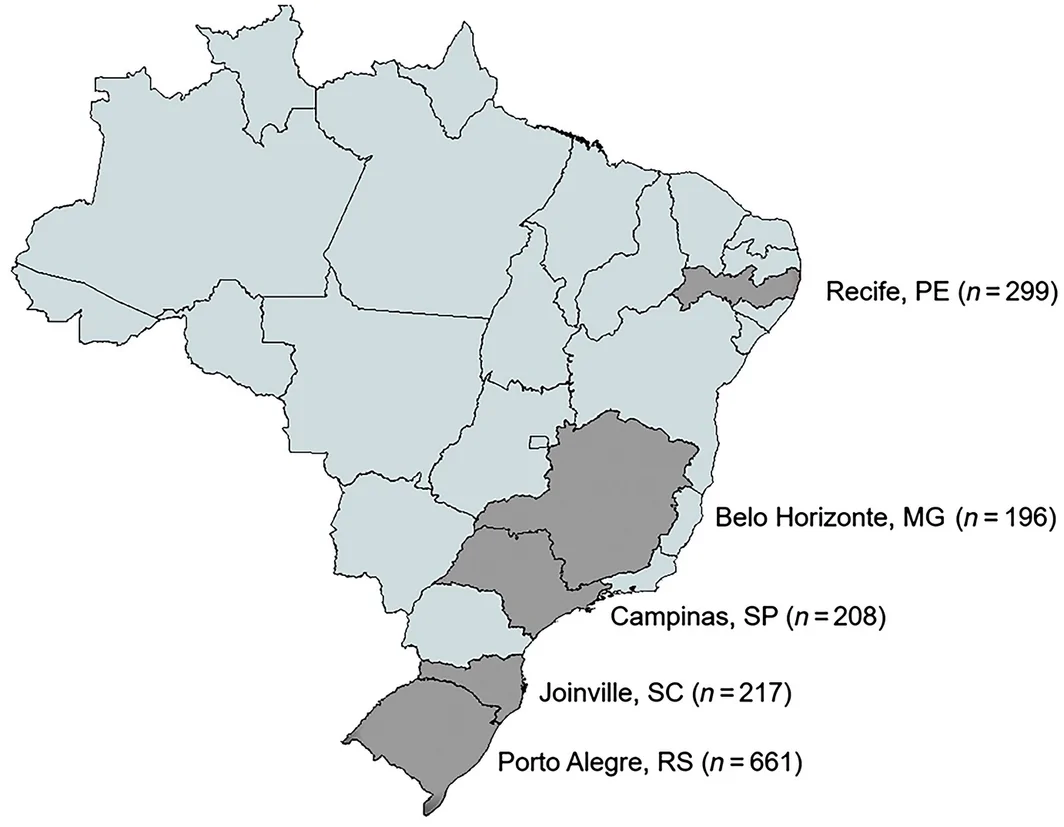
Geographical distribution of the centers included in this study.

Table 1 presents the demographic data of the sample, compiled according to the data available in each of the databases incorporated into this study. The mean age of participants ranged from 25 to 32 years across centers, with a median age in the overall sample of 28 years (interquartile range: 23–35 years). Among the 1385 women with parity information, 600 (43.3%) were primigravidas. Data on race/ethnicity were available for 960 women, of whom 542 (56.5%) self‐identified as white. The distribution by gestational age showed that 510 women (32.3%) had pregnancies beyond 37 weeks, 539 (34.1%) were between 34 and 36 + 6 weeks, and 532 (33.6%) were under 34 weeks. Regarding mode of delivery, 1065 records were available, of which 299 (28.1%) were vaginal births.

**TABLE 1:** Demographic characteristics of the sample included in this study, by total and by centers.

Center	Total	Porto Alegre	Recife	Campinas	Belo Horizonte	Joinville
Age (median, interquartile range, years)	28 [23–35]	28 [23–35]	25 [20–30]	29 [24–35]	30 [23.5–35]	32 [27–38]
Primigravida (*n =* 1385)	600 (43.3)	279 (42.2)	152 (50.8)	73 (35.1)	NA	96 (44.2)
Ethnicity (*n =* 960)	542 (56.5)	467 (70.7)	75 (25.1)	NA	NA	NA
White Other	418 (43.5)	194 (29.3)	224 (74.9)			
*Gestational age*						
>37 weeks	510 (32.3)	227 (35.2)	146 (48.8)	90 (43.3)	47 (24.0)	0
34–36 + 6	539 (34.1)	213 (31.8)	76 (25.4)	56 (26.9)	72 (36.7)	122 (56.2)
<34 weeks	532 (33.6)	221 (33.0)	77 (25.8)	62 (29.8)	77 (39.3)	95 (43.8)
Mode of delivery (*n =* 1065) Vaginal	299 (28.1%)	206 (31.2)	NA	43 (20.7)	50 (25.5)	NA

Abbreviation: NA, not available.

Table 2 shows that the median systolic blood pressure was 150 mmHg (IQR: 140–165), and the median diastolic pressure was 99 mmHg (IQR: 90–108). The fullPIERS score estimate had a median of 0.9% (IQR: 0.4%–2.0%) in the overall sample. Median values for laboratory test results are also presented in Table 2.

**TABLE 2:** Clinical and laboratorial characteristics of the sample included in this study, by total and by centers.

Center	Total	Porto Alegre	Recife	Campinas	Belo Horizonte	Joinville
Systolic blood pressure (mmHg)	150 [140–165]	147 [138–159]	160 [150–170]	150 [132–170]	150 [140–160]	157 [146–169]
Diastolic blood pressure (mmHg)	99 [90–108]	94 [87–102]	110 [100–110]	90 [80–100]	100 [90–110]	100 [92–110]
Blood oxygenation	98 [97–98]	98 [97–98]	98 [97–98]	97 [97–98]	98 [97–98]	98 [98–99]
Dispnea or chest pain	114 (7.2)	22 (3.3)	73 (24.4)	11 (5.3)	8 (4.1)	NA
FullPIERS	0.8 [0.4–2.0]	0.8 [0.3–1.7]	0.9 [0.4–2.6]	1.2 [0.5–2.4]	0.75 [0.4–2.3]	0.9 [0.3–1.8]
Creatinine	0.65 [0.57–0.75]	0.66 [0.58 0 0.79]	0.64 [0.57–0.69]	0.61 [0.52–0.75]	0.6 [0.55–0.7]	0.67 [0.6–0.76]
Transaminases	20 [15–27]	18 [15–26]	22 [17–30]	17 [14–22.5]	23 [18–32.5]	20 [16–30]
Platelets	200 [158–248]	200 [157–244]	213 [168–253]	193.5 [155–244]	211 [172.5–261.5]	186 [150–236]

*Note*: FullPiers, Pre‐eclampsia Integrated Estimate of Risk model.

Abbreviation: NA, not available.

Table 3 details the occurrence of each maternal outcome, both overall and by center. The most frequent severe outcome was HELLP syndrome, with 150 cases (9.5%), followed by placental abruption with 47 cases (3.0%). Two maternal deaths were recorded (0.1%). When analyzing results by center, HELLP syndrome was the leading complication associated with PE across all regions of Brazil, ranging from 6.6% to 19.9%, followed by eclampsia, which ranged from 1.0% to 3.8%.

**TABLE 3:** Adverse maternal outcomes of the sample included in this study, by total and by centers.

Center	Total	Porto Alegre	Recife	Campinas	Belo Horizonte	Joinville
HELLP	150 (9.5%)	44 (6.6%)	34 (11.4%)	14 (6.7%)	39 (19.9%)	19 (8.8%)
Eclampsia	36 (2.3%)	14 (2.1%)	3 (1.0%)	8 (3.8%)	5 (2.5%)	6 (2.8%)
Stroke	2 (0.1%)	1 (0.1%)	0	1 (0.5%)	0	0
Pulmonar edema	17 (1.1%)	11 (1.6%)	4 (1.3%)	1 (0.5%)	0	1 (0.4%)
Placental abruption	47 (3.0%)	34 (5.1%)	1 (0.3%)	1 (0.5%)	10 (5.1%)	1 (0.4%)
Maternal death	2 (0.1%)	0	0	1 (0.5%)	1 (0.5%)	0

Abbreviation: HELLP, hemolysis, elevated liver enzymes and low platelets.

Figure 2 presents the ROC curve for the use of fullPIERS in predicting severe maternal outcomes in the study sample, with an estimated AUC of 0.753 (95% confidence interval: 0.714–0.791), indicating a moderate predictive ability of the tool. The optimal cutoff point was 2.35%, with a sensitivity of 58.3% and specificity of 84.6%. Assuming a PE prevalence of 10%, the estimated accuracy was 82%, and the negative predictive value (NPV) was 94.8%.

**FIGURE 2:**
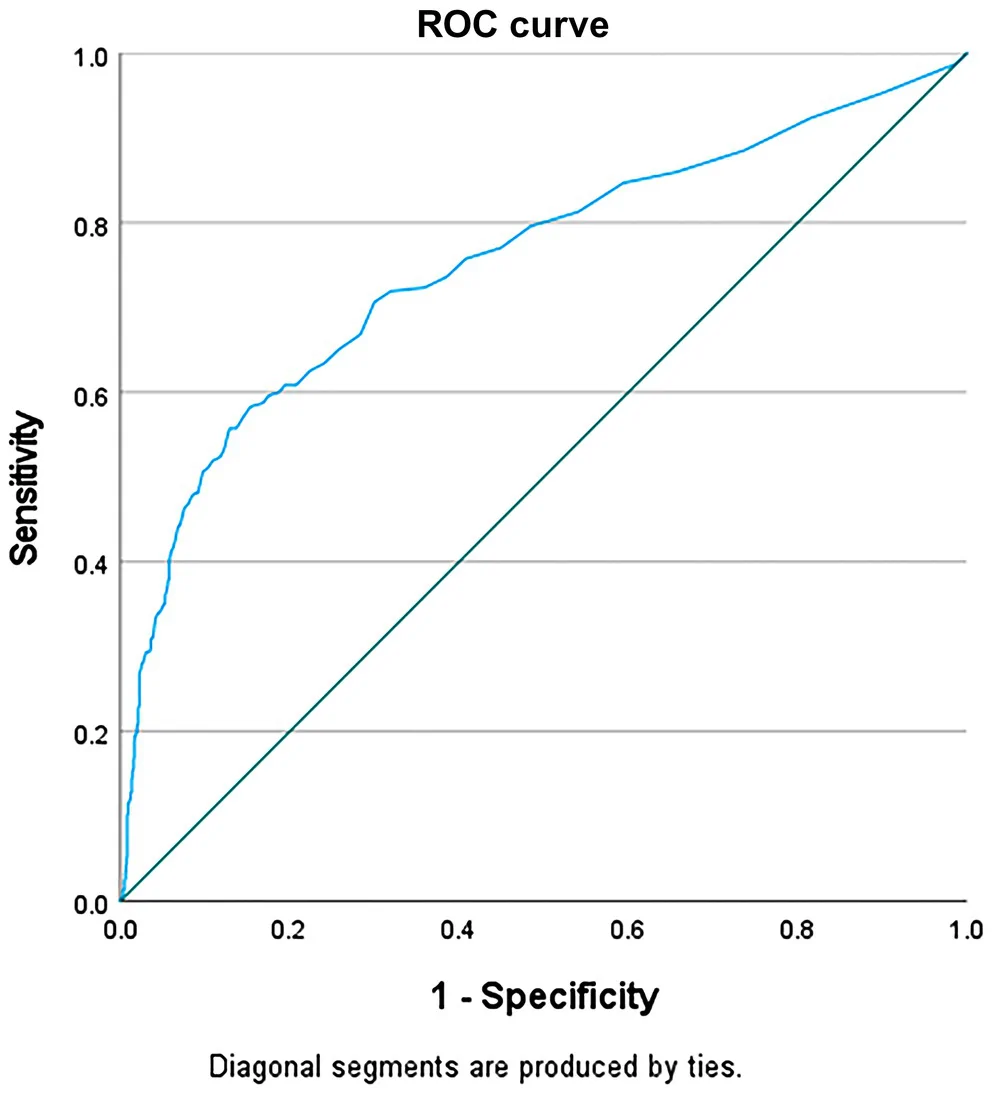
ROC curve for validation of fullPIERS in five Brazilian centers.

## 4 DISCUSSION

This study included 1581 Brazilian women with PE from referral obstetric centers across three different geographic regions of the country and estimated a prevalence of severe maternal outcomes of 14.86%. Using the fullPIERS model, it was found that values below 2.35% were effective in ruling out the occurrence of severe maternal outcomes, with an NPV of 94.8%.

This large, multicenter Brazilian validation of the fullPIERS model, encompassing five high‐complexity obstetric referral centers distributed across three distinct geographic regions, provides an important contribution to the global literature on prognostic models for PE. The findings reinforce the clinical utility of fullPIERS in stratifying maternal risk within 48 h of assessment, particularly in contexts where obstetric and neonatal care resources are limited. The relatively high proportion of early‐onset PE observed in our study reflects the tertiary referral characteristic of the participating institutions, which function as regional high‐complexity units and, therefore, receive a disproportionate number of severe and early‐onset cases. Consequently, the gestational age distribution reported here does not reflect population‐level incidence but, rather, the case mix typical of referral centers managing more complex presentations of the disease.

In countries with limited access to obstetric and neonatal care resources, it is essential to identify women who require hospital‐based care while also avoiding unnecessary admissions for those at lower risk of severe outcomes. The validation of the fullPIERS model in a large Brazilian dataset suggests that this tool has potential for broader application in the Brazilian context, considering its performance in terms of effectiveness and NPV.

In the original fullPIERS development study conducted in high‐income countries such as Canada, New Zealand, Australia, and the United Kingdom, the model achieved a high discriminatory capacity, with an AUC of 0.88 (95% CI: 0.84–0.92), combined with high specificity and an NPV.^7^ While the AUC in our study was lower (0.753), the specificity (0.846) and NPV (0.948) were quite similar, suggesting that in real‐world settings, as was the case in our population, the tool maintains good performance in identifying women at higher risk for severe maternal outcomes. This finding is of particular relevance for clinical decision‐making in resource‐constrained settings, where a reliable exclusion of imminent severe complications can help optimize the allocation of scarce hospital beds and specialized human resources.

Our findings are also in line with the external validation model of fullPIERS, which reported an AUC of 0.77 (95% CI: 0.72–0.82) and preserved good predictive performance in terms of NPV (72.2%).^14^ It is essential to highlight that the original model was developed in high‐income countries (Canada, New Zealand, Australia, and the UK). In contrast, the external validation study included data from Fiji, Uganda, South Africa, Brazil, and Pakistan.

In our study, HELLP syndrome was the most common manifestation of severity associated with PE, with a prevalence of 9.5%, consistently observed across the different participating centers. Placental abruption and eclampsia were less frequent. This underscores the importance of identifying women at risk for severe outcomes before clinical or laboratory manifestations become evident.

The present study also aligns with Latin American experiences, although with relevant distinctions in optimal cutoff values. A Mexican study validated the fullPIERS model in 130 women and found adequate discriminatory capacity (AUC of 0.718, 95% CI: 0.515–0.921). However, the optimal cutoff in that sample was 7.95%, which yielded a specificity of 99.2%.^15^ An exploratory analysis of our data showed that, at this same cutoff, although specificity increased to 97.5%, sensitivity dropped to 23.9%.

The good discriminatory performance of the fullPIERS model appears to be consistent across different settings, as evidenced by a US‐based study including 1510 participants, which reported an AUC of 0.80 (95% CI: 0.75–0.86). In that study, the best cutoff point was 5.5%, yielding a specificity of 85%.^16^ In contrast, a study from Peru with 367 participants reported a similar specificity with a much lower cutoff of 0.75% and a comparable AUC.^17^

These findings reinforce the need to validate the fullPIERS tool in different regions, taking into account the local prevalence of PE and the available capacity to provide care for the condition. A country's level of economic development influences its ability to offer adequate healthcare, and it seems that middle‐income countries might benefit from lower fullPIERS cutoffs, given the higher likelihood of severe maternal outcomes.

The applicability of the fullPIERS model in Brazil is particularly relevant given the regional disparities and structural limitations faced by many maternity units. In primary care facilities or small hospitals, the decision to refer a patient to a tertiary center is not always straightforward. In this context, a validated prognostic tool like fullPIERS can provide objective support to clinical decision‐making by identifying, at an early stage, women at higher risk of maternal deterioration. Beyond helping to rationalize the use of obstetric and neonatal beds, its implementation might prevent both delays in necessary referrals and unnecessary hospitalizations of low‐risk patients.^18^ This evaluation is especially relevant in regions where the distance between levels of care and the scarcity of resources deman safe decisions based on objective and reproducible criteria.^19^

It is essential that, beyond short‐term prediction of severe maternal outcomes, measures for prevention and immediate treatment be implemented. Among the preventive measures are low‐dose aspirin for women at increased risk of PE and calcium supplementation, particularly for populations with low dietary calcium intake, such as the Brazilian population.^20^, ^21^ Recently, Brazil's Ministry of Health adopted universal calcium supplementation as part of prenatal care, and the impact of this policy will be measurable in the coming years.

Additionally, healthcare professionals working in regions with high PE prevalence and complications must be trained to identify and treat the condition promptly. Training and availability of magnesium sulfate, a well‐established treatment for eclampsia, are also critical. The Brazilian Research Network on Hypertension in Pregnancy has conducted workshops across various regions of Brazil to train professionals in the use of magnesium sulfate.^22^

Although the fullPIERS model was developed to predict severe maternal outcomes, clinical decisions regarding the timing of delivery in PE invariably involve balancing maternal and fetal risks. The absence of fetal or neonatal outcomes in the model represents a limitation in real‐world clinical scenarios, where early delivery might prevent maternal complications but significantly increases neonatal morbidity and mortality. In practice, the decision to intervene often depends on the gestational age and estimated fetal well‐being, particularly in low‐resource settings where neonatal intensive care support might be limited or unavailable. Therefore, while fullPIERS offers valuable maternal risk stratification, its integration into clinical protocols should be accompanied by parallel assessment tools or strategies that consider fetal risks, to ensure comprehensive and context‐appropriate decision‐making.^23^

Among the strengths of this study, the most prominent is its multicentric nature, encompassing five referral obstetric centers from three distinct geographic regions of Brazil. This diversity in location, population characteristics, and healthcare delivery models enhances the external validity and generalizability of our findings. By including centers from the south, southeast, and northeast, we captured heterogeneity in sociodemographic profiles, disease severity at presentation, and institutional protocols, providing a more comprehensive picture of how the fullPIERS model performs in the Brazilian context. Previous national validations of fullPIERS^8^, ^9^, ^10^, ^11^ were limited in scope, either to single‐center samples or narrower geographic coverage, which restricted their applicability to the broader health system. The present work addresses that gap by integrating data from different epidemiological and care scenarios, more closely reflecting real‐world conditions.

Another strength lies in the representativeness of clinical presentations included. The cohort covered the full range of gestational ages and severity levels, from early‐onset PE with high maternal and perinatal risks to term cases where management decisions are more straightforward. This inclusivity ensures that the performance metrics reported here are applicable across the disease spectrum, which is particularly relevant for clinicians working in settings with unpredictable case mixes.

Despite these strengths, several limitations must be acknowledged. First, the retrospective nature of the analysis inherently limits control over the completeness and uniformity of data collection. Although rigorous procedures were used to identify and resolve inconsistencies, some variability between centers in data recording and laboratory measurement protocols is inevitable. Second, the data span a 10‐year period (2014–2024), during which clinical guidelines, resource availability, and referral patterns might have evolved. This temporal variation could have introduced heterogeneity in both case mix and management strategies. While this might be seen as a limitation, it also mirrors the dynamic nature of obstetric care in Brazil, potentially increasing the ecological validity of our findings.

Another limitation is the absence of fetal and neonatal outcomes in the fullPIERS model. In clinical practice, decisions regarding timing of delivery in PE require a delicate balance between maternal and perinatal risks.^19^ By focusing exclusively on maternal outcomes, the model might not fully capture the trade‐offs involved, especially in early gestations where neonatal morbidity and mortality risks are substantial. This limitation underscores the need for combined approaches, integrating fullPIERS with validated fetal assessment tools to inform comprehensive and individualized management plans.

Another important limitation of this study is that the 516 cases included do not have data on the mode of delivery. Those cases were included from centers where the delivery mode was not systematically recorded in the original datasets because this is not essential data for fullPIERS calculations. However, these cases were included because all data for the fullPIERS calculation and the occurrence of severe maternal outcomes were collected, and those outcomes were independent of the mode of delivery. We acknowledge that PE might determine the occurrence of a cesarean section, especially in countries with high rates of the procedure, such as Brazil^24^; however, the opposite is not true.

The determination of an appropriate cutoff is not merely a statistical exercise but a strategic clinical decision that must consider the epidemiology of severe maternal outcomes, the capacity of the health system to respond promptly to deterioration, and the potential harms of over‐ versus under‐triage. In Brazil, where there is a dual challenge of high PE burden and uneven distribution of specialized care, our findings support adopting a more conservative threshold to ensure that high‐risk cases are not overlooked.

In conclusion, the present multicenter Brazilian validation of the fullPIERS model demonstrates its potential as a practical, evidence‐based tool to support maternal risk assessment in PE. A cutoff of 2.35% achieved good discriminatory performance and a high NPV, aligning with the needs of a health system where timely identification of high‐risk patients is essential and resources are unevenly distributed. With further prospective validation and integration into national care protocols, fullPIERS could contribute significantly to optimizing maternal care pathways and improving outcomes in Brazil.

## 5 CONCLUSION

In this large, multicentre Brazilian validation, the fullPIERS model demonstrated good discriminatory performance and a high negative predictive value, supporting its role as a reliable decision support tool for maternal risk stratification in pre‐eclampsia. A conservative cutoff of 2.35% appears appropriate for the Brazilian context, balancing the need for clinical safety with the realities of limited and unevenly distributed healthcare resources.

By enabling the early identification of women at low short‐term risk, the model can support safe deferral of delivery in selected cases, potentially reducing unnecessary preterm births and optimizing the use of tertiary obstetric and neonatal resources. Conversely, by identifying those at highest risk of deterioration, it can inform timely referral and prioritization of care, particularly in underserved regions.

Prospective implementation studies across different levels of the health system are now warranted to assess its real‐world impact on referral patterns, intervention timing, and maternal and perinatal outcomes. With appropriate integration into national care protocols, fullPIERS has the potential to contribute meaningfully to reducing the burden of pre‐eclampsia and improving maternal health outcomes in Brazil.

## AUTHOR CONTRIBUTIONS

All authors participated in the original studies from which the present analysis was derived. JPG conceived and designed the study. All authors contributed their respective datasets. PVT performed the statistical analyses. JPG and PVT prepared the initial draft of the manuscript, which was critically revised for important intellectual content by MMA, LK, MLC, JM, GT, SHAMC, and JGLR. All authors contributed to the interpretation of the data, reviewed the manuscript for accuracy and clarity, and approved the final submitted version. All authors agree to be accountable for all aspects of the work, ensuring that questions related to the accuracy or integrity of any part of the work are appropriately investigated and resolved.

## CONFLICT OF INTEREST STATEMENT

The authors have no conflicts of interest to declare.

## Data Availability

The data that support the findings of this study are available on request from the corresponding author. The data are not publicly available due to privacy or ethical restrictions.
